# A case report of vasa previa incidentally discovered

**DOI:** 10.11604/pamj.2015.21.34.6697

**Published:** 2015-05-18

**Authors:** Salahiddine Saghir, Jaouad Kouach, Aomar Agadr

**Affiliations:** 1 Paediatric Department, Military Hospital Mohammed V, Rabat, Morocco; 2 Obstetrics and Gynecology Department, Military Hospital Mohammed V, Rabat, Morocco

**Keywords:** Vasa previa, fetal bleeding, Benckiser′s haemorrhage

## Abstract

Vasa previa is a rare but clinically important obstetrical complication that can be associated with a low-lying placenta or placenta previa. We aim to present one case of vasa previa diagnosed during the placenta examination after the caesarean indicated for triple uterus scar. A 26-year-old female was referred to our hospital at 30 weeks of gestation to provide a scheduled caesarean. Trans-abdominal ultrasound was performed; the placenta was positioned in the posterior side of the fundus. Fetal growth was found to be appropriate for gestational age. A healthy male infant weighing was successfully delivered via cesarean section at 38 weeks of gestation. This operation helped to prevent complications due to acute fetal bleeding. The identification and exclusion of vasa previa using trans-vaginal ultrasound are essential to ensure appropriate and timely treatment.

## Introduction

Vasa previa is a rare but clinically important obstetrical complication in which fetal blood vessels are positioned between the presenting part and cervix, and it can be associated with a low-lying placenta or placenta previa. The estimated incidence of vasa previa is approximately 1 in 2500 deliveries, but it is much higher (1 in 700) among patients who conceive through assisted reproductive technologies. The importance and clinical impact of an antenatal diagnosis of vasa previa is very significant because of the likelihood of adverse fetal outcomes [1]. Because the vessels are attached to the chorion, rupture of fetal membranes can result in fetal bleeding and death within minutes. When the condition is not diagnosed antenatally, the perinatal mortality rate is reported to be approximately 44%, whereas 97% of fetuses survive when an antenatal diagnosis is made, indicating significantly different outcomes [2, 3]. Furthermore, a low-lying placenta is reportedly a risk factor for vasa previa because it occurs in 5% of patients [4]. Accurate diagnosis of vasa previa is therefore crucial. Here, we describe a case of vasa previa incidentally discovered at the end of surgery.

## Patient and observation

**Figure 1:**
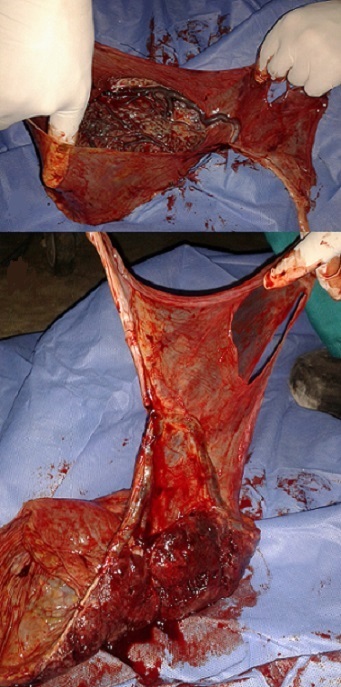
Placenta examination showing vasa previa

A 26-year-old female (gravida 4, para 3) was referred to our hospital at 30 weeks of gestation to provide a scheduled caesarean. Trans-abdominal ultrasound was performed; the placenta was positioned in the posterior side of the fundus. Fetal growth was found to be appropriate for gestational age. The patient was admitted at 38 weeks of gestation to the surgery room according to the guidelines for the management of the uterine multiple scars. A healthy male infant weighing was successfully delivered via cesarean section, the Apgar score was 9/10/10 with no blood loss during delivery. The evaluation of the placenta confirmed the diagnosis of vasa previa (Figure 1). The postoperative course of the patient and the infant was uncomplicated and they were discharged 3 days after delivery in a healthy condition.

## Discussion

Previously, vasa previa was usually detected by palpation of the fetal vessels within the membranes during labor or on the basis of acute-onset vaginal bleeding and subsequent fetal bradycardia and/or death after membrane rupture. As discussed above, the importance of an accurate diagnosis of vasa previa is significant; if not diagnosed antenatally, the neonatal survival rate is only 44% with a neonatal transfusion rate of 58.5% [2]. A universal screening method for the detection of vasa previa has not yet been established [5] although high-risk factors have been identified [6]. Baulies et al. reported that the incidence of vasa previa was 0.07%, and multivariate analysis revealed the following associated factors in their study. In vitro fertilization (IVF) pregnancies, Therefore, if patients present with any of these risk factors, a concerted effort to detect vasa previa using ultrasound screening in the second trimester is necessary [7]. Screening for high-risk patients (such as those with IVF pregnancies, a velamentous cord, a low-lying placenta, low cord insertions in the uterus, or a low-lying bilobate placenta) has shown some success [1, 8, 9]. The primary methods of diagnosis are trans-vaginal ultrasonography and real-time color Doppler ultrasonography, and most cases are diagnosed antenatally. Unfortunately, in our case detailed ultrasonography in the second trimester for the screening of vasa previa and to detect the cord insertion was not performed; therefore, it was difficult to make diagnosis of vasa previa.

## Conclusion

We presented a case of vasa previa diagnosed postoperatively during caesarean scheduled for uterine multiple scars, which helped to prevent complications due to acute fetal bleeding. The identification and exclusion of vasa previa using trans-vaginal ultrasound are essential to ensure appropriate and timely treatment.
